# *N*,*N*′-Dibenzyl-*N*,*N*′-dimethyl-*N*′′-(4-nitro­benzo­yl)phospho­ric triamide

**DOI:** 10.1107/S1600536811011275

**Published:** 2011-04-07

**Authors:** Mehrdad Pourayoubi, Mahnaz Rostami Chaijan, Laura Torre-Fernández, Santiago García-Granda

**Affiliations:** a Department of Chemistry, Ferdowsi University of Mashhad, Mashhad, 91779, Iran; b Departamento de Química Física y Analítica, Facultad de Química, Universidad de Oviedo - CINN C/ Julián Clavería, 8, 33006 Oviedo (Asturias) Spain

## Abstract

The P atom in the title compound, C_23_H_25_N_4_O_4_P, is in a slightly distorted tetra­hedral coordination environment and the N atoms show *sp* ^2^ character. The phosphoryl group and the NH unit are *syn* with respect to each other. In the crystal, pairs of inter­molecular N—H⋯O(P) hydrogen bonds form centrosymmetric dimers.

## Related literature

For phospho­rus compounds with general formula *XP*(O)[N(CH_3_)(CH_2_C_6_H_5_)]_2_, see: Gholivand *et al.* (2005 ▶). For bond lengths in a related structure, see: Sabbaghi *et al.* (2010 ▶). For hydrogen-bond motifs, see: Etter *et al.* (1990 ▶); Bernstein *et al.* (1995 ▶).
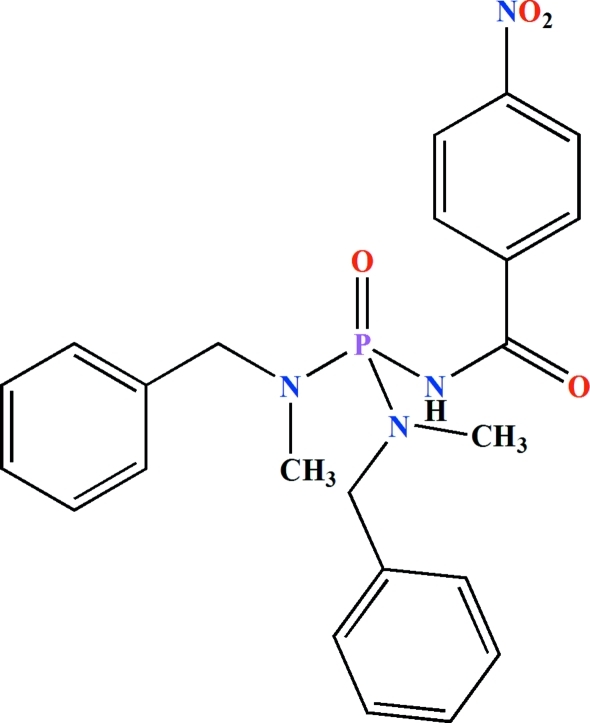


## Experimental

### 

#### Crystal data

C_23_H_25_N_4_O_4_P
*M* *_r_* = 452.44
Triclinic, 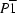

*a* = 8.3526 (5) Å
*b* = 11.8150 (5) Å
*c* = 12.2668 (4) Å
α = 77.184 (3)°
β = 81.289 (4)°
γ = 71.928 (4)°
*V* = 1117.70 (9) Å^3^
*Z* = 2
Cu *K*α radiation
μ = 1.41 mm^−1^
*T* = 297 K
0.24 × 0.14 × 0.05 mm

#### Data collection

Oxford Diffraction Xcalibur Ruby Gemini diffractometer
Absorption correction: multi-scan (*CrysAlis PRO*; Oxford Diffraction, 2010 ▶) *T*_min_ = 0.941, *T*_max_ = 1.000
8669 measured reflections
4203 independent reflections
3779 reflections with *I* > 2σ(*I*)
*R*_int_ = 0.025

#### Refinement

*R*[*F* ^2^ > 2σ(*F* ^2^)] = 0.035
*wR*(*F* ^2^) = 0.093
*S* = 1.05
4203 reflections
390 parameters
All H-atom parameters refined
Δρ_max_ = 0.21 e Å^−3^
Δρ_min_ = −0.26 e Å^−3^

### 

Data collection: *CrysAlis PRO* (Oxford Diffraction, 2010 ▶); cell refinement: *CrysAlis PRO*; data reduction: *CrysAlis PRO*; program(s) used to solve structure: *SIR2004* (Burla *et al.*, 2005 ▶); program(s) used to refine structure: *SHELXL97* (Sheldrick, 2008 ▶); molecular graphics: *Mercury* (Macrae *et al.*, 2008 ▶); software used to prepare material for publication: *WinGX* (Farrugia, 1999 ▶).

## Supplementary Material

Crystal structure: contains datablocks global, I. DOI: 10.1107/S1600536811011275/ng5139sup1.cif

Structure factors: contains datablocks I. DOI: 10.1107/S1600536811011275/ng5139Isup2.hkl

Additional supplementary materials:  crystallographic information; 3D view; checkCIF report

## Figures and Tables

**Table 1:** Hydrogen-bond geometry (Å, °)

*D*—H⋯*A*	*D*—H	H⋯*A*	*D*⋯*A*	*D*—H⋯*A*
N8—H8⋯O2^i^	0.85 (2)	2.07 (2)	2.909 (2)	169 (2)

Symmetry code: (i) 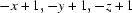.

**Fig. 1.**
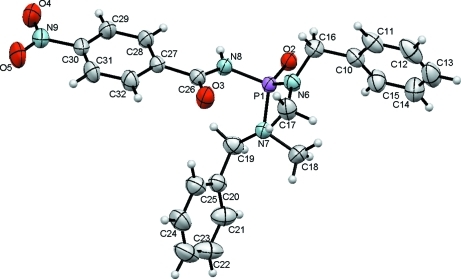
An ORTEP style plot of title compound with the atom-labeling scheme. Ellipsoids are shown at the 50% probability level. H atoms are represented as small spheres of arbitrary radii.

## supplementary crystallographic information

### Comment

Some phosphoric triamide compounds of the general formula *XP*(O)[N(CH_3_)(CH_2_C_6_H_5_)]_2_ [*X* = Cl, C_6_H_5_C(O)NH & CCl_3_C(O)NH (Gholivand *et al.*, 2005) have been structurally investigated. Here, we report on the synthesis and crystal structure of title compound (where *X* = 4-NO_2_C_6_H_4_C(O)NH).

The asymmetric unit consists of a single molecule, shown in Fig. 1, of the title compound with no unusual bonding features. The P═O and P—N bond lengths are comparable to those in similar compounds like for example in P(O)[NHC(O)C_6_H_4_(4-NO_2_)][NHC_6_H_11_]_2_ (Sabbaghi *et al.*, 2010). As can be expected, the N8—C26 bond length is shorter than the other N—C bonds in the molecule.

The phosphorus atom has a slightly distorted tetrahedral configuration, the bond angles around the P atom are in the range of 103.85 (6)° [N6—P1—N7] to 118.67 (6)° [O2—P1—N7]. The average of surrounding angles around the tertiary nitrogen atom N6 (119.7°) shows that it is bonded in an essentially planar geometry; whereas, the environment of N7 is slightly deviated from planarity (average of bond angles around N7 atom is equal to 117.3°). Furthermore, the angle C26—N8—P1 is 124.98 (10)°.

The oxygen atom of P═O group is a better H-acceptor than that of the C═ O counterpart; so, the hydrogen atom of the C(O)NHP(O) moiety is involving in an intermolecular –P═O···H—N– hydrogen bond (see Table 1) to form a centrosymmetric dimer [graph set: *R*_2_^2^(8) (Etter *et al.*, 1990; Bernstein *et al.*, 1995)].

### Experimental

4-NO_2_—C_6_H_4_C(O)NHP(O)Cl_2_ was prepared according to the procedure of literature (Sabbaghi *et al.*, 2010). To a solution of (2 mmol) 4-NO_2_C_6_H_4_C(O)NHP(O)Cl_2_ in CH_3_CN (20 ml), a solution of *N*-methylbenzyl amine (8 mmol) in CH_3_CN (5 ml) was added dropwise at 273 K. After 4 h stirring, the solvent was removed in vacuum. Single crystals were obtained from a solution of title compound in C_2_H_5_OH after slow evaporation at room temperature. IR (KBr, cm^-1^): 3141, 2881, 1680, 1604, 1522, 1452, 1342, 1273, 1186, 1104, 1005, 949, 853, 793, 708.

### Refinement

At the end of the refinement the highest peak in the electron density was 0.210 e Å ^-3^, while the deepest hole was -0.260 e Å ^-3^. All H atoms were sucessfully located by difference Fourier synthesis and isotropically refined.

### Crystal data

**Table:**

C_23_H_25_N_4_O_4_P	*Z* = 2
*M**_r_* = 452.44	*F*(000) = 476
Triclinic, *P*1	*D*_x_ = 1.344 Mg m^−^^3^
Hall symbol: -P 1	Cu *K*α radiation, λ = 1.54180 Å
*a* = 8.3526 (5) Å	Cell parameters from 5608 reflections
*b* = 11.8150 (5) Å	θ = 3.7–70.5°
*c* = 12.2668 (4) Å	µ = 1.41 mm^−^^1^
α = 77.184 (3)°	*T* = 297 K
β = 81.289 (4)°	Prismatic, colorless
γ = 71.928 (4)°	0.24 × 0.14 × 0.05 mm
*V* = 1117.70 (9) Å^3^	

### Data collection

**Table:**

Oxford Diffraction Xcalibur Ruby Gemini diffractometer	4203 independent reflections
Radiation source: Enhance (Cu) X-ray Source	3779 reflections with *I* > 2σ(*I*)
graphite	*R*_int_ = 0.025
Detector resolution: 10.2673 pixels mm^-1^	θ_max_ = 70.6°, θ_min_ = 3.7°
ω scans	*h* = −10→10
Absorption correction: multi-scan (*CrysAlis PRO*; Oxford Diffraction, 2010)	*k* = −12→14
*T*_min_ = 0.941, *T*_max_ = 1.000	*l* = −14→14
8669 measured reflections	

### Refinement

**Table:**

Refinement on *F*^2^	Secondary atom site location: difference Fourier map
Least-squares matrix: full	Hydrogen site location: inferred from neighbouring sites
*R*[*F*^2^ > 2σ(*F*^2^)] = 0.035	All H-atom parameters refined
*wR*(*F*^2^) = 0.093	*w* = 1/[σ^2^(*F*_o_^2^) + (0.049*P*)^2^ + 0.1381*P*] where *P* = (*F*_o_^2^ + 2*F*_c_^2^)/3
*S* = 1.05	(Δ/σ)_max_ = 0.001
4203 reflections	Δρ_max_ = 0.21 e Å^−^^3^
390 parameters	Δρ_min_ = −0.26 e Å^−^^3^
0 restraints	Extinction correction: *SHELXL*, Fc^*^=kFc[1+0.001xFc^2^λ^3^/sin(2θ)]^-1/4^
Primary atom site location: structure-invariant direct methods	Extinction coefficient: 0.0073 (5)

### Special details

**Table:**

Geometry. All e.s.d.'s (except the e.s.d. in the dihedral angle between two l.s. planes) are estimated using the full covariance matrix. The cell e.s.d.'s are taken into account individually in the estimation of e.s.d.'s in distances, angles and torsion angles; correlations between e.s.d.'s in cell parameters are only used when they are defined by crystal symmetry. An approximate (isotropic) treatment of cell e.s.d.'s is used for estimating e.s.d.'s involving l.s. planes.
Refinement. Refinement of *F*^2^ against ALL reflections. The weighted *R*-factor *wR* and goodness of fit *S* are based on *F*^2^, conventional *R*-factors *R* are based on *F*, with *F* set to zero for negative *F*^2^. The threshold expression of *F*^2^ > σ(*F*^2^) is used only for calculating *R*-factors(gt) *etc*. and is not relevant to the choice of reflections for refinement. *R*-factors based on *F*^2^ are statistically about twice as large as those based on *F*, and *R*- factors based on ALL data will be even larger.

### Fractional atomic coordinates and isotropic or equivalent isotropic displacement parameters (Å^2^)

**Table:**

	*x*	*y*	*z*	*U*_iso_*/*U*_eq_	
P1	0.37947 (4)	0.43216 (3)	0.68154 (3)	0.03514 (12)	
O2	0.39241 (13)	0.55008 (8)	0.61457 (8)	0.0441 (2)	
O3	0.40102 (16)	0.16957 (10)	0.70306 (10)	0.0570 (3)	
O4	1.0449 (2)	−0.09851 (14)	0.30155 (13)	0.0866 (5)	
O5	0.9874 (2)	−0.24087 (12)	0.42737 (14)	0.0867 (5)	
N6	0.18214 (15)	0.43519 (10)	0.71762 (10)	0.0422 (3)	
N7	0.46802 (15)	0.38121 (10)	0.80040 (10)	0.0415 (3)	
N8	0.48310 (16)	0.32916 (10)	0.59984 (10)	0.0404 (3)	
N9	0.9666 (2)	−0.13399 (13)	0.38622 (13)	0.0594 (4)	
C10	−0.08796 (18)	0.59948 (13)	0.70707 (13)	0.0455 (3)	
C11	−0.2562 (2)	0.61378 (15)	0.69736 (16)	0.0559 (4)	
C12	−0.3838 (2)	0.69259 (18)	0.7548 (2)	0.0693 (6)	
C13	−0.3441 (3)	0.75656 (19)	0.82172 (19)	0.0718 (6)	
C14	−0.1776 (3)	0.7439 (2)	0.8315 (2)	0.0717 (5)	
C15	−0.0499 (2)	0.66649 (16)	0.77376 (17)	0.0587 (4)	
C16	0.0512 (2)	0.51473 (15)	0.64516 (14)	0.0494 (4)	
C17	0.1226 (2)	0.34870 (15)	0.80655 (16)	0.0537 (4)	
C18	0.3946 (3)	0.44794 (17)	0.89219 (14)	0.0563 (4)	
C19	0.6514 (2)	0.32321 (15)	0.80428 (14)	0.0484 (3)	
C20	0.69385 (18)	0.22357 (14)	0.90560 (12)	0.0451 (3)	
C21	0.7774 (2)	0.2365 (2)	0.98905 (15)	0.0615 (4)	
C22	0.8215 (3)	0.1426 (3)	1.08008 (17)	0.0796 (6)	
C23	0.7830 (3)	0.0367 (2)	1.08917 (18)	0.0742 (6)	
C24	0.7001 (3)	0.02349 (18)	1.00715 (18)	0.0697 (5)	
C25	0.6558 (3)	0.11582 (16)	0.91598 (16)	0.0592 (4)	
C26	0.48804 (18)	0.20940 (12)	0.62439 (11)	0.0402 (3)	
C27	0.61200 (18)	0.12548 (12)	0.55456 (11)	0.0392 (3)	
C28	0.7086 (2)	0.16247 (13)	0.45913 (12)	0.0478 (4)	
C29	0.8241 (2)	0.07733 (14)	0.40268 (13)	0.0526 (4)	
C30	0.8402 (2)	−0.04361 (13)	0.44417 (13)	0.0461 (3)	
C31	0.7468 (2)	−0.08292 (14)	0.53841 (15)	0.0543 (4)	
C32	0.6313 (2)	0.00252 (14)	0.59370 (15)	0.0515 (4)	
H11	−0.278 (3)	0.569 (2)	0.6490 (18)	0.069 (6)*	
H12	−0.499 (4)	0.701 (2)	0.745 (2)	0.092 (7)*	
H13	−0.432 (3)	0.809 (2)	0.860 (2)	0.087 (7)*	
H14	−0.154 (3)	0.786 (2)	0.882 (2)	0.093 (8)*	
H15	0.067 (3)	0.6575 (19)	0.7821 (18)	0.070 (6)*	
H16A	0.104 (2)	0.5613 (17)	0.5845 (17)	0.054 (5)*	
H16B	0.001 (3)	0.4617 (19)	0.6165 (17)	0.061 (5)*	
H17A	0.041 (3)	0.3951 (19)	0.8615 (18)	0.067 (6)*	
H17B	0.218 (3)	0.292 (2)	0.843 (2)	0.076 (6)*	
H17C	0.070 (3)	0.303 (2)	0.7758 (19)	0.076 (6)*	
H18A	0.276 (3)	0.490 (2)	0.885 (2)	0.084 (7)*	
H18B	0.444 (3)	0.512 (3)	0.892 (2)	0.096 (8)*	
H18C	0.407 (3)	0.394 (2)	0.964 (2)	0.088 (7)*	
H19A	0.702 (3)	0.3836 (19)	0.8089 (17)	0.061 (5)*	
H19B	0.693 (2)	0.2926 (17)	0.7372 (17)	0.057 (5)*	
H21	0.803 (3)	0.312 (2)	0.9826 (19)	0.074 (6)*	
H22	0.878 (4)	0.154 (3)	1.137 (3)	0.117 (10)*	
H23	0.810 (3)	−0.029 (3)	1.148 (2)	0.096 (8)*	
H24	0.672 (3)	−0.051 (2)	1.009 (2)	0.078 (6)*	
H25	0.600 (3)	0.1054 (19)	0.8612 (19)	0.069 (6)*	
H28	0.698 (2)	0.2437 (19)	0.4309 (17)	0.061 (5)*	
H29	0.894 (3)	0.101 (2)	0.3376 (19)	0.070 (6)*	
H31	0.766 (3)	−0.165 (2)	0.5660 (18)	0.069 (6)*	
H32	0.565 (3)	−0.0228 (19)	0.6597 (18)	0.066 (6)*	
H8	0.533 (2)	0.3600 (17)	0.5403 (17)	0.049 (5)*	

### Atomic displacement parameters (Å^2^)

**Table:**

	*U*^11^	*U*^22^	*U*^33^	*U*^12^	*U*^13^	*U*^23^
P1	0.0438 (2)	0.02572 (18)	0.03071 (18)	−0.00681 (13)	0.00261 (13)	−0.00327 (12)
O2	0.0586 (6)	0.0298 (5)	0.0380 (5)	−0.0110 (4)	0.0061 (4)	−0.0035 (4)
O3	0.0742 (7)	0.0362 (5)	0.0535 (6)	−0.0178 (5)	0.0162 (5)	−0.0060 (5)
O4	0.0973 (11)	0.0691 (9)	0.0630 (9)	0.0154 (8)	0.0114 (8)	−0.0189 (7)
O5	0.1171 (13)	0.0377 (7)	0.0854 (10)	0.0103 (7)	−0.0052 (9)	−0.0200 (7)
N6	0.0434 (6)	0.0352 (6)	0.0402 (6)	−0.0065 (5)	0.0002 (5)	−0.0006 (5)
N7	0.0460 (6)	0.0371 (6)	0.0354 (6)	−0.0048 (5)	−0.0009 (5)	−0.0064 (5)
N8	0.0530 (7)	0.0292 (5)	0.0337 (6)	−0.0098 (5)	0.0060 (5)	−0.0044 (4)
N9	0.0701 (9)	0.0463 (8)	0.0522 (8)	0.0072 (6)	−0.0137 (7)	−0.0185 (6)
C10	0.0418 (7)	0.0386 (7)	0.0505 (8)	−0.0079 (6)	−0.0075 (6)	0.0004 (6)
C11	0.0500 (9)	0.0452 (8)	0.0674 (10)	−0.0159 (7)	−0.0141 (8)	0.0087 (8)
C12	0.0398 (9)	0.0600 (11)	0.0866 (14)	−0.0080 (8)	−0.0010 (8)	0.0178 (10)
C13	0.0578 (11)	0.0591 (11)	0.0732 (12)	0.0025 (9)	0.0135 (9)	−0.0011 (10)
C14	0.0686 (12)	0.0629 (11)	0.0768 (13)	−0.0026 (9)	−0.0034 (10)	−0.0243 (10)
C15	0.0469 (9)	0.0555 (10)	0.0723 (11)	−0.0054 (7)	−0.0089 (8)	−0.0198 (8)
C16	0.0514 (8)	0.0469 (8)	0.0468 (8)	−0.0072 (7)	−0.0101 (7)	−0.0086 (7)
C17	0.0516 (9)	0.0425 (8)	0.0578 (10)	−0.0134 (7)	0.0105 (8)	−0.0012 (7)
C18	0.0698 (11)	0.0528 (10)	0.0400 (8)	−0.0051 (8)	−0.0041 (7)	−0.0146 (7)
C19	0.0449 (8)	0.0500 (9)	0.0451 (8)	−0.0141 (7)	−0.0012 (6)	0.0005 (7)
C20	0.0377 (7)	0.0486 (8)	0.0418 (7)	−0.0068 (6)	−0.0007 (5)	−0.0036 (6)
C21	0.0593 (10)	0.0786 (13)	0.0509 (9)	−0.0310 (9)	−0.0070 (7)	−0.0029 (8)
C22	0.0693 (12)	0.1179 (19)	0.0511 (10)	−0.0358 (12)	−0.0214 (9)	0.0091 (11)
C23	0.0635 (11)	0.0782 (14)	0.0599 (11)	−0.0073 (10)	−0.0144 (9)	0.0174 (10)
C24	0.0835 (14)	0.0454 (10)	0.0674 (12)	−0.0087 (9)	−0.0101 (10)	0.0039 (8)
C25	0.0723 (11)	0.0496 (9)	0.0524 (9)	−0.0128 (8)	−0.0143 (8)	−0.0037 (7)
C26	0.0502 (8)	0.0306 (6)	0.0365 (7)	−0.0097 (6)	−0.0020 (6)	−0.0034 (5)
C27	0.0493 (7)	0.0294 (6)	0.0370 (7)	−0.0073 (5)	−0.0076 (6)	−0.0056 (5)
C28	0.0675 (10)	0.0283 (7)	0.0388 (7)	−0.0052 (6)	−0.0007 (7)	−0.0035 (6)
C29	0.0698 (10)	0.0393 (8)	0.0377 (7)	−0.0044 (7)	0.0022 (7)	−0.0058 (6)
C30	0.0548 (8)	0.0353 (7)	0.0434 (8)	0.0017 (6)	−0.0126 (6)	−0.0128 (6)
C31	0.0673 (10)	0.0279 (7)	0.0617 (10)	−0.0059 (7)	−0.0064 (8)	−0.0069 (7)
C32	0.0605 (9)	0.0320 (7)	0.0558 (9)	−0.0105 (6)	0.0018 (7)	−0.0042 (6)

### Geometric parameters (Å, °)

**Table:**

P1—O2	1.4787 (10)	C17—H17C	0.95 (2)
P1—N6	1.6319 (13)	C18—H18A	0.97 (3)
P1—N7	1.6420 (12)	C18—H18B	0.96 (3)
P1—N8	1.6910 (11)	C18—H18C	0.97 (3)
O3—C26	1.2163 (18)	C19—C20	1.510 (2)
O4—N9	1.206 (2)	C19—H19A	0.95 (2)
O5—N9	1.219 (2)	C19—H19B	0.95 (2)
N6—C17	1.462 (2)	C20—C25	1.380 (3)
N6—C16	1.4684 (19)	C20—C21	1.382 (2)
N7—C18	1.4663 (19)	C21—C22	1.391 (3)
N7—C19	1.474 (2)	C21—H21	0.96 (2)
N8—C26	1.3686 (18)	C22—C23	1.364 (4)
N8—H8	0.85 (2)	C22—H22	0.96 (3)
N9—C30	1.4729 (19)	C23—C24	1.365 (3)
C10—C11	1.383 (2)	C23—H23	0.93 (3)
C10—C15	1.385 (2)	C24—C25	1.383 (3)
C10—C16	1.509 (2)	C24—H24	0.97 (2)
C11—C12	1.395 (3)	C25—H25	0.92 (2)
C11—H11	0.95 (2)	C26—C27	1.5035 (19)
C12—C13	1.367 (3)	C27—C28	1.385 (2)
C12—H12	0.96 (3)	C27—C32	1.391 (2)
C13—C14	1.373 (3)	C28—C29	1.388 (2)
C13—H13	0.94 (3)	C28—H28	0.93 (2)
C14—C15	1.389 (3)	C29—C30	1.377 (2)
C14—H14	0.96 (3)	C29—H29	0.96 (2)
C15—H15	0.97 (2)	C30—C31	1.369 (2)
C16—H16A	0.96 (2)	C31—C32	1.383 (2)
C16—H16B	1.00 (2)	C31—H31	0.92 (2)
C17—H17A	1.01 (2)	C32—H32	0.96 (2)
C17—H17B	0.96 (2)		
			
O2—P1—N6	110.89 (6)	H18A—C18—H18B	104 (2)
O2—P1—N7	118.67 (6)	N7—C18—H18C	110.9 (15)
N6—P1—N7	103.85 (6)	H18A—C18—H18C	110 (2)
O2—P1—N8	105.24 (6)	H18B—C18—H18C	108 (2)
N6—P1—N8	113.52 (6)	N7—C19—C20	112.83 (12)
N7—P1—N8	104.82 (6)	N7—C19—H19A	107.8 (12)
C17—N6—C16	113.89 (13)	C20—C19—H19A	107.4 (12)
C17—N6—P1	125.48 (11)	N7—C19—H19B	108.0 (11)
C16—N6—P1	119.62 (10)	C20—C19—H19B	110.6 (12)
C18—N7—C19	112.51 (13)	H19A—C19—H19B	110.2 (17)
C18—N7—P1	117.29 (10)	C25—C20—C21	118.07 (16)
C19—N7—P1	122.11 (10)	C25—C20—C19	121.29 (15)
C26—N8—P1	124.98 (10)	C21—C20—C19	120.60 (16)
C26—N8—H8	122.7 (12)	C20—C21—C22	120.3 (2)
P1—N8—H8	112.4 (12)	C20—C21—H21	118.3 (14)
O4—N9—O5	123.56 (15)	C22—C21—H21	121.4 (14)
O4—N9—C30	118.42 (14)	C23—C22—C21	121.0 (2)
O5—N9—C30	118.01 (16)	C23—C22—H22	120.3 (19)
C11—C10—C15	118.31 (16)	C21—C22—H22	118.7 (19)
C11—C10—C16	121.02 (15)	C22—C23—C24	119.00 (19)
C15—C10—C16	120.65 (14)	C22—C23—H23	124.1 (17)
C10—C11—C12	120.54 (18)	C24—C23—H23	116.9 (17)
C10—C11—H11	116.3 (13)	C23—C24—C25	120.7 (2)
C12—C11—H11	123.1 (13)	C23—C24—H24	121.8 (14)
C13—C12—C11	120.40 (18)	C25—C24—H24	117.5 (14)
C13—C12—H12	121.6 (16)	C20—C25—C24	120.95 (19)
C11—C12—H12	118.0 (16)	C20—C25—H25	119.4 (13)
C12—C13—C14	119.66 (19)	C24—C25—H25	119.6 (14)
C12—C13—H13	119.4 (15)	O3—C26—N8	121.84 (13)
C14—C13—H13	120.9 (16)	O3—C26—C27	120.11 (12)
C13—C14—C15	120.3 (2)	N8—C26—C27	117.97 (12)
C13—C14—H14	117.8 (16)	C28—C27—C32	119.72 (13)
C15—C14—H14	121.8 (17)	C28—C27—C26	124.63 (12)
C10—C15—C14	120.80 (18)	C32—C27—C26	115.62 (13)
C10—C15—H15	119.7 (13)	C27—C28—C29	120.19 (14)
C14—C15—H15	119.5 (13)	C27—C28—H28	121.6 (12)
N6—C16—C10	112.55 (12)	C29—C28—H28	118.2 (12)
N6—C16—H16A	108.2 (11)	C30—C29—C28	118.48 (15)
C10—C16—H16A	109.3 (11)	C30—C29—H29	120.0 (13)
N6—C16—H16B	107.3 (12)	C28—C29—H29	121.5 (13)
C10—C16—H16B	108.7 (12)	C31—C30—C29	122.66 (14)
H16A—C16—H16B	110.8 (16)	C31—C30—N9	118.90 (14)
N6—C17—H17A	108.4 (12)	C29—C30—N9	118.42 (15)
N6—C17—H17B	109.0 (14)	C30—C31—C32	118.49 (14)
H17A—C17—H17B	110.9 (18)	C30—C31—H31	120.1 (13)
N6—C17—H17C	110.5 (14)	C32—C31—H31	121.3 (14)
H17A—C17—H17C	110.8 (18)	C31—C32—C27	120.47 (16)
H17B—C17—H17C	107 (2)	C31—C32—H32	120.0 (13)
N7—C18—H18A	110.9 (15)	C27—C32—H32	119.6 (13)
N7—C18—H18B	113.1 (17)		
			
O2—P1—N6—C17	−162.93 (13)	N7—C19—C20—C21	−112.67 (17)
N7—P1—N6—C17	−34.39 (15)	C25—C20—C21—C22	0.2 (3)
N8—P1—N6—C17	78.85 (14)	C19—C20—C21—C22	−177.67 (17)
O2—P1—N6—C16	29.27 (13)	C20—C21—C22—C23	−0.2 (3)
N7—P1—N6—C16	157.80 (11)	C21—C22—C23—C24	0.1 (3)
N8—P1—N6—C16	−88.96 (12)	C22—C23—C24—C25	0.1 (3)
O2—P1—N7—C18	67.60 (14)	C21—C20—C25—C24	−0.1 (3)
N6—P1—N7—C18	−56.00 (14)	C19—C20—C25—C24	177.82 (17)
N8—P1—N7—C18	−175.36 (13)	C23—C24—C25—C20	−0.1 (3)
O2—P1—N7—C19	−78.73 (13)	P1—N8—C26—O3	8.3 (2)
N6—P1—N7—C19	157.67 (12)	P1—N8—C26—C27	−168.53 (10)
N8—P1—N7—C19	38.30 (13)	O3—C26—C27—C28	175.52 (15)
O2—P1—N8—C26	−173.12 (12)	N8—C26—C27—C28	−7.6 (2)
N6—P1—N8—C26	−51.67 (14)	O3—C26—C27—C32	−6.7 (2)
N7—P1—N8—C26	60.98 (14)	N8—C26—C27—C32	170.15 (14)
C15—C10—C11—C12	−0.9 (2)	C32—C27—C28—C29	0.0 (2)
C16—C10—C11—C12	−179.86 (15)	C26—C27—C28—C29	177.69 (15)
C10—C11—C12—C13	−0.2 (3)	C27—C28—C29—C30	−0.3 (3)
C11—C12—C13—C14	0.6 (3)	C28—C29—C30—C31	0.2 (3)
C12—C13—C14—C15	−0.1 (3)	C28—C29—C30—N9	−178.13 (15)
C11—C10—C15—C14	1.5 (3)	O4—N9—C30—C31	177.97 (17)
C16—C10—C15—C14	−179.55 (18)	O5—N9—C30—C31	−2.8 (2)
C13—C14—C15—C10	−1.0 (3)	O4—N9—C30—C29	−3.7 (2)
C17—N6—C16—C10	65.91 (18)	O5—N9—C30—C29	175.58 (17)
P1—N6—C16—C10	−124.93 (13)	C29—C30—C31—C32	0.2 (3)
C11—C10—C16—N6	−132.44 (15)	N9—C30—C31—C32	178.54 (15)
C15—C10—C16—N6	48.6 (2)	C30—C31—C32—C27	−0.5 (3)
C18—N7—C19—C20	66.18 (18)	C28—C27—C32—C31	0.4 (2)
P1—N7—C19—C20	−146.04 (12)	C26—C27—C32—C31	−177.47 (15)
N7—C19—C20—C25	69.5 (2)		

### Hydrogen-bond geometry (Å, °)

**Table:**

*D*—H···*A*	*D*—H	H···*A*	*D*···*A*	*D*—H···*A*
N8—H8···O2^i^	0.85 (2)	2.07 (2)	2.909 (2)	169 (2)

Symmetry codes: (i) −*x*+1, −*y*+1, −*z*+1.

## References

[bb1] Bernstein, J., Davis, R. E., Shimoni, L. & Chang, N.-L. (1995). *Angew. Chem. Int. Ed. Engl.* **34**, 1555–1573.

[bb2] Burla, M. C., Caliandro, R., Camalli, M., Carrozzini, B., Cascarano, G. L., De Caro, L., Giacovazzo, C., Polidori, G. & Spagna, R. (2005). *J. Appl. Cryst.* **38**, 381–388.

[bb3] Etter, M. C., MacDonald, J. C. & Bernstein, J. (1990). *Acta Cryst.* B**46**, 256–262. 10.1107/s0108768189012929 2344397

[bb4] Farrugia, L. J. (1999). *J. Appl. Cryst.* **32**, 837–838.

[bb5] Gholivand, K., Pourayoubi, M., Shariatinia, Z. & Mostaanzadeh, H. (2005). *Polyhedron*, **24**, 655–662.

[bb6] Macrae, C. F., Bruno, I. J., Chisholm, J. A., Edgington, P. R., McCabe, P., Pidcock, E., Rodriguez-Monge, L., Taylor, R., van de Streek, J. & Wood, P. A. (2008). *J. Appl. Cryst.* **41**, 466–470.

[bb7] Oxford Diffraction (2010). *CrysAlis PRO* Oxford Diffraction Ltd, Abingdon, England.

[bb8] Sabbaghi, F., Pourayoubi, M., Toghraee, M. & Divjakovic, V. (2010). *Acta Cryst.* E**66**, o344. 10.1107/S1600536810000851 PMC2979854 21579772

[bb9] Sheldrick, G. M. (2008). *Acta Cryst.* A**64**, 112–122. 10.1107/S0108767307043930 18156677

